# Susac syndrome—a rare rheumatology mimic

**DOI:** 10.1093/rap/rkac020

**Published:** 2022-03-21

**Authors:** Anuoluwapo R Oke, Jyotin Pandit, Azeem Ahmed

**Affiliations:** 1 Rheumatology Department; 2 Ophthalmology Department, Great Western Hospital, Swindon, UK

Key message

- Susac syndrome symptoms overlap with various rheumatic diseases; awareness is crucial for prompt Multidisciplinary team collaboration.

Dear Editor, A 26-year-old female with intermittent numbness and tingling in both hands later developed symptoms of migraine with visual aura in September 2019. She experienced recurrent severe episodes of vertigo, nausea, vomiting associated with ataxic gait and horizontal nystagmus. Initially, she was suspected to have labyrinthitis. She had no past medical history or family history.

Nine months after her initial symptoms, she developed fluctuating hearing loss in her right ear, with continuous low- and occasional high-pitched tinnitus. ENT examination revealed nystagmus on lateral gaze with a negative Romberg’s and Unterberg’s test. Audiology examination showed a right-side sensorineural hearing loss at low frequency in her right ear (40 dB at 250 Hz) and normal findings in her left ear (Fig. 1A). Tympanograms were normal.

**Fig. 1:**
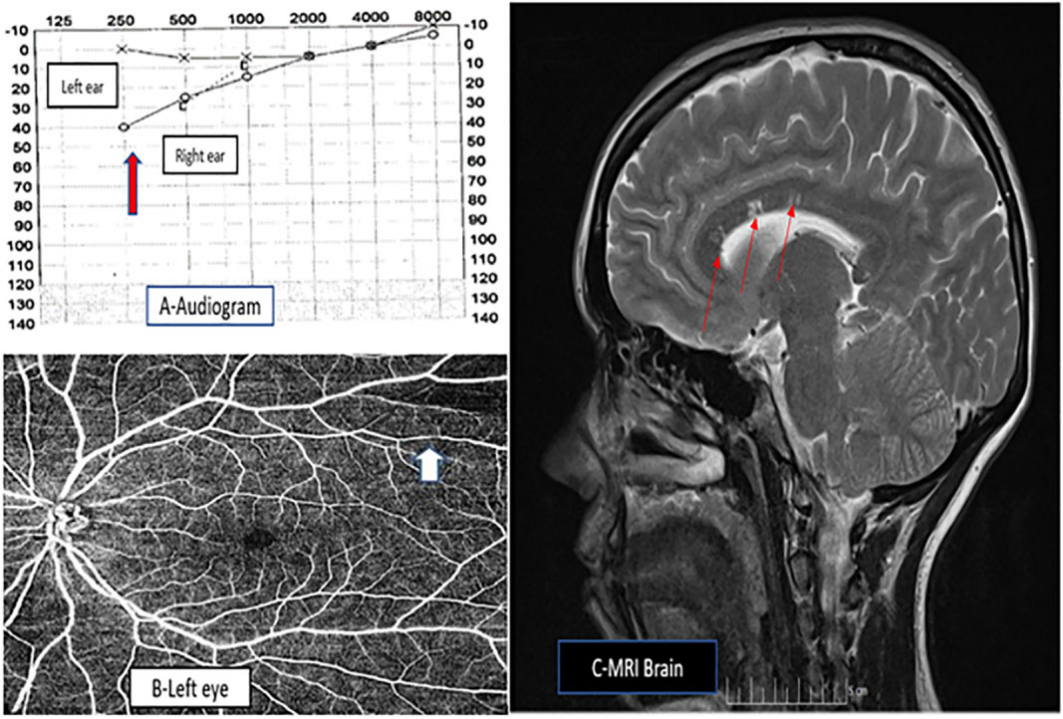
Susac syndrome (**A**) Audiogram showing sensorineural hearing loss at low frequency in right ear. (**B**) Optical Coherence Tomography angiogram showing superotemporal branch retinal artery occlusion in the left eye. (**C**) MRI brain sagittal view showing snowball lesions on T2 images.

She later presented to the eye department with sudden onset painless visual field defect in her left eye. Ophthalmic examination revealed left superotemporal branch retinal artery occlusion, which was confirmed on Optical Coherence Tomography angiogram (Fig. 1B). Blood investigation showed normal CRP (0.2 mg/L), ESR (2 mm in 1 hr), RF < 10 IU/mL, negative aPL screen (Beta 2 Gp-1, ACL, lupus anticoagulant), ANCA, syphilis, Lyme, and hepatitis serology and a negative CTD screen. A lumbar puncture showed an oligoclonal band in the cerebrospinal fluid and serum consistent with a systemic immune response. Her cerebrospinal fluid protein was 0.32 g/l (normal range: 0.15–0.45 g/l) with glucose of 3.8 mmol/l (normal range: 2.2–3.9 mmol/l).

An urgent MRI scan of her head showed no evidence of acoustic neuroma and revealed multiple lesions in her corpus callosum that represent micro-infarcts (Fig. 1C). She was diagnosed with Susac syndrome based on her symptoms and these findings.

She was treated with a 5-day course of i.v. methylprednisolone (500 mg daily) and subsequently commenced on 60 mg of prednisolone on a reducing course. She has made good improvement and is currently on MMF 1 g twice daily and low-dose prednisolone, with stabilization and no further worsening of her symptoms.

Susac syndrome is a rare form of micro-angiopathy and is thought to be an autoimmune endotheliopathy, with CD8^+^ T cells implicated in its pathogenesis [1]. The exact aetiology is unknown.

The true incidence and prevalence are unknown. It has no racial predilection. Females are often affected (female:male ratio of 3:1), and the syndrome commonly occurs within the second to fourth decade of life [2, 3]. The course of the disease can be monocyclic, polycyclic and sometimes fluctuates or can be chronic and continuous [3, 4].

It is characterized by a triad of encephalopathy, branch retinal artery occlusion and inner ear disease, notably hearing loss. The symptom triad often appears in successive intervals over years, and only ∼13% of patients develop the triad at presentation [5]. Peripheral numbness is rarely reported in Susac syndrome. It was the initial presenting feature in our patient and has been described in a few cases [6]. Other symptoms include recurrent headaches, dysarthria, memory impairment, confusion, personality/behavioural disturbances, tinnitus, sensorineural hearing loss and visual loss, with development of dark spots.

Systemic rheumatology diseases, including Behçet’s disease, Cogan’s syndrome, large and small vessel vasculitis, CNS vasculitis, SLEs and relapsing polychondritis, are possible mimics of this rare syndrome. Other important differentials include multiple sclerosis, given the female preponderance and age of onset, Meniere’s disease, Lyme’s disease, encephalitis and stroke.

A complete neurology, audiology and ophthalmology examination is essential to aid diagnosis. A fluorescein angiogram is essential in suspected cases and often shows retinal arteriole wall hyperfluorescence, gas plaques sometimes with vessel leakage [7]. Audiogram often reveals low- to mid-frequency sensorineural hearing loss. MRI of the brain is the neuroimaging modality of choice. This shows characteristic T2 hyperintensity changes in the central portion of the corpus callosum, termed snowballs, which are best seen on sagittal T2 FLAIR images (Fig. 1C). Cerebrospinal fluid examination often shows a high protein level, with presence of oligoclonal band.

Early and aggressive treatment is crucial to prevent irreversible damage. Given that it is a rare disease, no consensus on effective therapy is available. The mainstay of treatment is the use of CS. In addition, immunosuppressive therapy, including CYC, IVIG, AZA and MMF, anti-TNF, rituximab and anti-thrombotic therapy are useful. Cochlear implant can be considered in severe hearing impairment.

*Funding:* No specific funding was received from any funding bodies in the public, commercial or not-for-profit sectors to carry out the work described in this manuscript.

*Disclosure statement:* The authors have declared no conflicts of interest.

*Consent:* The patient provided informed consent for the publication of this manuscript.

## Data availability statement

Data are available upon reasonable request by any qualified researchers who engage in rigorous, independent scientific research, and will be provided following review and approval of a research proposal and Statistical Analysis Plan (SAP) and execution of a Data Sharing Agreement (DSA). All data relevant to the study are included in the article.
